# Adolescent cannabis use and young adult healthcare use in a population-based birth cohort linked to healthcare administrative records

**DOI:** 10.1192/j.eurpsy.2025.359

**Published:** 2025-08-26

**Authors:** P. Martinez, M.-C. Geoffroy, C. Temcheff, S. Côté, R. Tremblay, M. Boivin, M. Orri

**Affiliations:** 1 Department of Psychiatry, McGill Group for Suicide Studies, Douglas Mental Health University Institute; 2 Department of Educational and Counselling Psychology, McGill University; 3 École de Santé Publique, University of Montreal, Montreal; 4 Research Unit on Children’s Psychosocial Maladjustment, Québec, Canada; 5 School of Public Health, Physiotherapy, and Sports Science, University College Dublin, Dublin, Ireland; 6 Department of Psychology, Université Laval, Quebec; 7 Department of Psychiatry, McGill Group for Suicide Studies, Douglas Mental Health University Institute, Montreal, Canada

## Abstract

**Introduction:**

Evidence links early adolescent cannabis use (CU) to long-term health risks, but most studies lack comprehensive early-life confounder data and rely on subjective health measures.

**Objectives:**

To assess the association between adolescent CU trajectories and healthcare use for physical and mental health problems (P&MHP) in young adulthood.

**Methods:**

Data from the Québec Longitudinal Study of Child Development, a 23-year population-based birth cohort (N = 1,591), were linked to healthcare administrative records (hospitalizations, outpatient, and ER visits). CU trajectories (exposure) were derived from age of onset and frequency data (ages 12-17) using group-based trajectory modeling. Missing data on pre-exposure confounders were multiply imputed. Overlap-weighted logistic regression was used to assess the adjusted associations between these trajectories and healthcare use for P&MHP between ages 18-23.

**Results:**

Three CU trajectories were identified: non-users, late users, and early users (Figure 1). Early users had a higher risk of healthcare use for any mental disorder (OR 1.55, 95% CI 1.17-2.06), common mental disorders (OR 1.69, 95% CI 1.19-2.39), substance-related disorders (OR 2.25, 95% 1.24-4.10), and hospitalizations for physical diseases (OR 1.57, 95% CI 1.03-2.38) compared to non-users. No significant differences were found between late and non-users.

**Image 1:**

**Figure:**
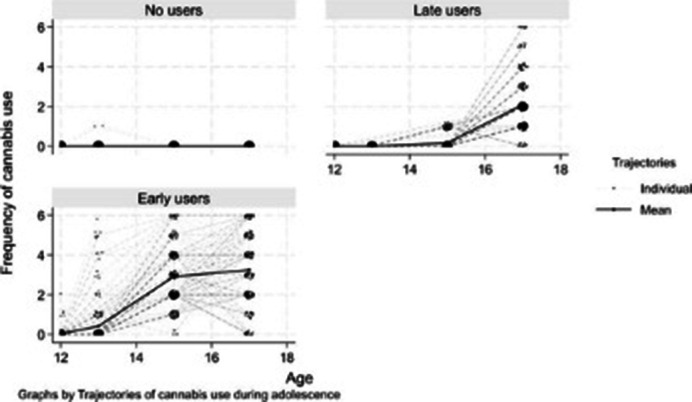

**Conclusions:**

These findings highlight the need for targeted interventions during adolescence to mitigate long-term health risks. Prevention efforts should prioritize early users, and be focused on integrated social, mental, and physical care.

**Disclosure of Interest:**

None Declared

